# Six Weeks of Core Stability Training Improves Landing Kinetics Among Female Capoeira Athletes: A Pilot Study

**DOI:** 10.1515/hukin-2015-0004

**Published:** 2015-04-07

**Authors:** Simone Araujo, Daniel Cohen, Lawrence Hayes

**Affiliations:** 1 School of Human Science, London Metropolitan Universit, London, UK.; 2 Instituto de Investigaciones, Escuela de Medicina, Universidad de Santander, Bucaramanga, Santander, Colombia.; 3 Institute of Clinical Exercise and Health Science, University of the West of Scotland.

**Keywords:** exercise training, drop jump, injury prevention, female athletes, injury risk

## Abstract

Core stability training (CST) has increased in popularity among athletes and the general fitness population despite limited evidence CST programmes alone lead to improved athletic performance. In female athletes, neuromuscular training combining balance training and trunk and hip/pelvis dominant CST is suggested to reduce injury risk, and specifically peak vertical ground reaction forces (vGRF) in a drop jump landing task. However, the isolated effect of trunk dominant core stability training on vGRF during landing in female athletes had not been evaluated. Therefore, the objective of this study was to evaluate landing kinetics during a drop jump test following a CST intervention in female capoeira athletes. After giving their informed written consent, sixteen female capoeira athletes (mean ± SD age, stature, and body mass of 27.3 ± 3.7 years, 165.0 ± 4.0 cm, and 59.7 ± 6.3 kg, respectively) volunteered to participate in the training program which consisted of static and dynamic CST sessions, three times per week for six weeks. The repeated measures T-test revealed participants significantly reduced relative vGRF from pre- to post-intervention for the first (3.40 ± 0.78 vs. 2.85 ± 0.52 N·NBW-1, respectively [p<0.05, effect size = 0.60]), and second landing phase (5.09 ± 1.17 vs. 3.02 ± 0.41 N·NBW-1, respectively [p<0.001, effect size = 0.87]). The average loading rate was reduced from pre- to post-intervention during the second landing phase (30.96 ± 18.84 vs. 12.06 ± 9.83 N·NBW·s-1, respectively [p<0.01, effect size = 0.68]). The peak loading rate was reduced from pre- to post-intervention during the first (220.26 ± 111.51 vs. 120.27 ± 64.57 N·NBW·s-1 respectively [p<0.01, effect size = 0.64]), and second (99.52 ± 54.98 vs. 44.71 ± 30.34 N·NBW·s-1 respectively [p<0.01, effect size = 0.70]) landing phase. Body weight, average loading rate during the first landing phase, and jump height were not significantly different between week 0 and week 6 (p=0.528, p=0.261, and p=0.877, respectively). This study provides evidence that trunk dominant core stability training improves landing kinetics without improving jump height, and may reduce lower extremity injury risk in female athletes.

## Introduction

Core stability (CS) refers to musculature control around the lumbo-pelvic region, with the aim of maintaining functional stability in a neutral position and assisting in the generation and transfer of energy from the trunk to the extremities (Akuthota and Nadler, 2004; Shirey et al., 2012). CS is important to athletes and recreationally active individuals alike as it provides proximal stability for distal mobility, especially in cases involving spinal stability (Akuthota et al., 2008; Panjabi, 1992). CS training (CST) is used extensively in injury prevention and rehabilitation, but more recently it is also being used as a means to enhance sports performance (Akuthota and Nadler, 2004; Sato and Mokha, 2009; Liemohn et al., 2005).

Numerous training and sports activities involve jumps and require appropriate jump landing technique. Jump landing technique and peak landing forces during a drop jump test have been intimated as potential indicators of injury risk to the lower limbs (McNair et al., 2000; Yeow et al., 2010). There is strong evidence that females have greater peak landing forces per kilogram of body mass than males, and are more likely to sustain a non-contact lower extremity injury when landing from a jump (Seegmiller and McCaw, 2003; Shirey et al., 2012; Yeow et al., 2010). High vertical ground reaction forces on landing are associated with high anterior cruciate ligament (ACL) loading and with ACL injury in prospective studies (McNair and Marshall, 1994; Yu et al., 2006). The higher relative landing forces in females are thought to contribute to their higher injury risk (Hewett et al., 2006).

There is some evidence that CST is effective at improving athletic performance (Ford et al., 2003). Dynamic stability of the trunk and lower limbs are based on the neuromuscular control of the lumbo-pelvic-hip complex. This complex consists of the hip, pelvis and trunk segment, as well as the muscles that cross these joints (Hibbs et al., 2008; Okada et al., 2011; Oliver et al., 2012). CST has previously improved stability and endurance capacity of the core musculature (Ekstrom et al., 2007; Fredericson and Moore, 2005; Imai et al., 2010), which may explain improved performance in endurance events (Sato and Mokha, 2009).

Females have a greater risk of lower limb injury than males (Barber-Westin et al., 2010; Myer et al., 2006; Shirey et al., 2012). Additionally, associations between poor core stability of the trunk and non-contact anterior cruciate ligament (ACL) injuries in female athletes have been described (Hewett et al., 2006; Leetun et al., 2004; Zazulak et al., 2007). Specifically, poor core neuromuscular control may increase external hip adduction and knee valgus moments during landing (Leetun et al., 2004) which increases ACL loading (Shin et al., 2011). Landings are common in gymnastics, and are the cause of many injuries, possibly due to the high ground reaction force (GRF) observed in these activities (Seegmiller and McCaw, 2003). Capoeira is a Brazilian martial art which combines balance, agility and strength, and involves acrobatic and dance movements as well as jumping and landing movements, similar to gymnastics (Assuncao, 2005). As such these athletes may also be at a higher risk of lower extremity injuries due to frequent exposure to high landing forces.

It is proposed that dynamic knee stability depends on core control (Shirey et al., 2012), and there is some epidemiological evidence showing an association between poorer neuromuscular control of the trunk and ACL injuries (Zazulak et al., 2007). Furthermore, Myer and colleagues (2006) reported that a neuromuscular training program which included both balance and mainly dynamic core stability exercises for the trunk and pelvis significantly reduced impact landing forces, whilst plyometric training did not. While this evidence suggests incorporation of CS exercises to a training routine can reduce peak landing forces and may also lead to a decrease in injury risk, it is unclear what the specific impact of a trunk dominant CST intervention alone is.

As previous work has evaluated the first landing phase of the drop jump (landing from the box), but it has been proposed that the second landing phase (after the maximal jump) may be more relevant to sports situations (Bates et al., 2013), both landing phases were of interest in this study. Therefore, the aim of the present pilot investigation was to establish whether a six week training intervention of trunk dominant CST would improve landing kinetics in the first and second landing phase of a drop jump in female capoeira athletes.

## Material and Methods

### Participants

Sixteen female capoeira athletes with a mean ± standard deviation (SD) age, stature, and body mass of 27.3 ± 3.7 years, 165.0 ± 4.0 cm, and 59.7 ± 6.3 kg, respectively, volunteered to participate in the study. Participants had a minimum of two years capoeira training and reported being free from injury in the last six months. Experimental procedures were approved by the London Metropolitan University Ethics Committee. The protocol was explained and participants gave their written informed consent to participate in this study and completed a physical activity readiness questionnaire (PAR-Q). All participants were asked to abstain from alcohol, caffeine and vigorous exercise for the 24 h preceding assessment at week 0 and week 6.

### Procedures

After several initial familiarisation trials, participants reported to the laboratory for pre-intervention assessment (week 0) of drop jump performance and measurement of height and body mass. The same procedure was repeated six weeks later (week 6), after the completion of the intervention. Participants were well trained but not involved in a periodized training programme and therefore acted as their own controls. Participants were allocated the same appointment time to avoid the influence of diurnal variation in neuromuscular performance (Hayes et al., 2010). Following a standardised warm up, participants performed three drop jump tests (Ford et al., 2003) in bare feet from a wooden box with a height of 40 cm. The box was placed on the floor 10 cm in front of a force platform with a sampling frequency of 1000 Hz (Kistler 5211, Kistler, Winterhur, Switzerland) consistent with previous investigations (Ford et al., 2003; McNair et al., 2000; Shultz and Schmitz, 2009). For each jump, participants were given a “3-2-1” verbal countdown and instructed to drop directly onto the force platform and immediately perform a maximum vertical jump, keeping their hands on their hips to eliminate any variability attributed to arm swing. Instructions were given to employ the same technique for each jump separated by 1 min rest intervals to limit neuromuscular fatigue and ensure consistency between participants. Peak landing force was defined as the highest vertical GRF (vGRF) observed following the drop from the box (landing phase one) and following the maximal vertical jump (landing phase two). Participants performed three trials and the mean vGRF was calculated for the first landing phase and the second landing phase. Relative peak landing force (N·N_BW_^−1^) was calculated by dividing the peak landing force (N) by participants body weight (N). In addition, average loading rate (N·N_BW_·s^−1^) during each landing phase was calculated using equation 1.

Equation 1 $$\text{Average}\;\text{loading}\;\text{rate}\;(1^{\text{st}}\;\text{landing}\;\text{phase})=\frac{\left(\text{peak}\;\text{vGRF}-\text{vGRF}\;\text{at}\;\text{first}\;\text{foot}\;\text{contact}\right)}{\text{time}\;\text{interval}}$$

The peak loading rate was calculated as the steepest vGRF slope at any point during the time interval in equation 1. Vertical jump height was provided by computer software (BioWare, Edmonton, Alberta, Canada) using equation 2 defined by Linthorne (2001).

Equation 2 $$\text{Jump}\;\text{height}=\frac{{\left(9.81-\text{flight}\;\text{time}+2\right)}^{2}}{9.81^{2}}$$

To avoid experimenter bias, no specific instructions were provided with regard to landing mechanics. A recorded jump was discarded and repeated if participants lost their balance, did not land bilaterally, moved their hands from their hips, or failed to land on the force platform after the vertical jump. The training intervention is displayed in Table 1 and all training sessions were supervised by a member of the research team and conducted prior to regular capoeira training. Exercises were demonstrated by the first author and technique coaching was provided if required.

### Statistical Analysis

Data were analysed using SPSS (version 20.0, IBM North America, New York, NY, USA). Once parametric assumptions were met, repeated measures T-tests were used to test for differences in landing forces, loading rates, body weight, and jump height pre- versus post-intervention. Significance was set *a priori* at p <0.05 and data are reported as mean ± SD. Effect size (Cohen, 1988) is reported where appropriate and effect sizes of 0.2, 0.5, and 0.8 were classed as small, medium, and large, respectively. Statistical power for the sample size used (n=16) was 1.00.

## Results

After the six week intervention, peak vGRF was reduced by 0.55 N·N_BW_^−1^ (p<0.05, effect size = 0.60) during the first landing phase and by 2.09 N·N_BW_^−1^ (p<0.001, effect size = 0.87) during the second landing phase (Table 2). This represents a 16% and 41% reduction in peak VGRF during the landing phase one and two, respectively. The average loading rate was reduced during the second landing phase by 18.9 N·N_BW_·s^−1^ (p<0.01, effect size = 0.68) from pre- to post-intervention which represents a 61% reduction. The peak loading rate was reduced by 99.99 N·N_BW_·s^−1^ (p<0.01, effect size = 0.64) during the first landing phase, and by 54.81 N·N_BW_·s^−1^ (p<0.01, effect size = 0.70) during the second landing phase, which represents a 55% reduction during both phases. Body weight, average loading rate during the first landing phase, and jump height were not significantly different between week 0 and week 6 (p=0.528, p=0.261, and p=0.877, respectively).

## Discussion

The purpose of this study was to evaluate the effectiveness of a six week CST programme on landing kinetics during a drop jump in female capoeira athletes. After the intervention, peak vGRF during the first and second landing phase of the drop jump was reduced compared to pre-intervention. To our knowledge, the present investigation was the first to evaluate the effect of a CST program with substantial proportion of static exercises, on peak vGRF assessed during a drop jump. Iida et al. (2013) reported that a 2 week landing training program led to a significant 19% decrease in peak landing force per kg body mass, in the first landing phase compared to a 1.4% decrease in a control group, with no change in jump height. We observed a large (41%; effect size 0.87), significant decrease in second landing phase peak vGRF. In cross-sectional and intervention studies that examined landing forces in the drop jump in relation to injury risk, vGRF during the first landing phase has typically been evaluated, however, it is proposed that the second landing phase is more representative of sports/training situation (Bates et al., 2013). Bates et al. (2013) argued that due to the intervening maximal jump and the shift in focus from landing there is greater perturbation and a more unstable body posture in-flight in the second than the first landing phase. Therefore, the decreased vGRF in the second landing phase may be as, or even more important as those observed in the first landing phase. Our finding of a significant decrease in loading rates, may be equally, if not more relevant than peak values. Dissipating a given amount of force over shorter time duration is thought to increase mechanical strain on a joint and is associated with ACL injury risk (Hewett et al., 2005).

Sato and Mokha (2009) reported no significant influence of a CST intervention on vGRF measured by running over a force platform, rather than from a jump. Nevertheless, there were improvements in 500 m run times, indicating that CST significantly improved sports performance. In contrast, we observed no improvement in vertical jump height as a result of the intervention, which is consistent with other studies (Schiling et al., 2013; Parkhouse and Ball, 2011). However, unlike the present investigation, exercises investigated by Sato and Mokha (2009) consisted mainly of dynamic contractions, without emphasis on sustained isometric contractions such as the plank, known to activate deep abdominal musculature (Stanton et al., 2004). Exercises presented in Sato and Mokha’s (2009) investigation could also be regarded as relatively novice (Stanton et al., 2004).

Ekstrom et al. (2007) assessed electromyography (EMG) in the plank and side plank and concluded these exercises produced sufficient activation of trunk muscles such as the rectus abdominus, multifidus, and external oblique to stimulate adaptations in muscular endurance of these muscles. Moreover, Schilling et al. (2013) reported significant improvements in measures of flexor and lateral endurance following six weeks of training that included the side plank and one other isometric exercise indicating that the acute activation profile was reflected in measurable trunk muscular endurance adaptations. Whilst it is unclear why improvements in muscular endurance would alter trunk activation in such a way as to alter landing mechanics under non-fatiguing conditions, there is evidence that these static trunk exercises do result in improvement in stability of the trunk and lower extremity during jump landings. Imai and colleagues (2014) reported that in youth soccer players, a program that included static exercises such as the plank and side plank but not a “conventional program” of dynamic flexion and extension exercises led to significant improvements in the rebound jump performance (flight time/contact time) and suggested these exercises improved control of the trunk position during landing impact. Thus, while muscular endurance tends to be the outcome measure of performance in the trunk, due to methodological difficulties in assessment of maximal isometric strength in these muscles, it is not unreasonable to speculate that some improvements in maximal strength were also stimulated (Kraemer and Ratamess, 2004). Indeed, Hides et al. (2012) reported that in elite Australian Rules footballers abdominal drawing-in exercises (sustained isometric contractions of the trunk) aimed to develop endurance of deep abdominal and back muscles provided sufficient overload to promote significant increases in the cross-sectional area of the multifidus.

Whilst we cannot fully expound the mechanism by which the CST intervention reduced peak vGRF during a drop jump, it has previously been shown that poor neuromuscular control of the trunk is associated with increased valgus, abduction motion and torque at the knee (Hewett et al., 2005) and lower extremity injury incidence (Zazulak et al., 2007) and that this kinematic profile is associated with higher peak vGRF (Hewett et al., 1999). Conversely, neuromuscular training that included training of the trunk reduced knee abduction torques and tendency to valgus collapse during landing (Myer et al., 2006). Trunk muscle activity precedes activity of lower extremity musculature and the position and movement of the trunk during landing has a substantial influence on ground reaction force (Kulas et al., 2006). Forward inclination of the trunk during landing for example, was reported to decrease peak vGRF (Blackburn and Padua, 2009). Increased intra-abdominal pressure is observed prior to ground contact and trunk muscles are activated in feedforward postural control in preparation for absorption of landing impact (Kulas et al., 2006). Co-contraction of trunk extensors and flexors as well as lower extremity muscles increase stiffness and protect joint structures of lower limbs such that a larger magnitude of impact forces during landing increases trunk stiffness (Horita et al., 2002). Iida et al. (2011) reported that impact force was positively correlated with the % EMG of maximum voluntary contraction of the rectus abdominus muscle. These authors suggested during landing, the increased activation of rectus abdominus and co-contractions of trunk extensors produced greater intra-abdominal pressure and increased trunk stiffness were responsible for increased vGRF on landing. On this basis, we speculated that decreased vGRF following the training intervention may be related to a reduction in the % of maximum EMG required due to an increase in maximal strength capabilities. Our findings may also have been mediated by changes in trunk strength producing alterations in landing posture, which is a determinant of landing vGRF (Kulas et al., 2006). Further work is needed to evaluate the interaction between trunk muscle strength and landing vGRF and kinematics.

Based on previous evidence among female athletes, reduction in peak landing force observed in the present investigation could reduce knee injury risk (Myer et al., 2006; Hewett et al., 1999). Peak vGRF is strongly correlated with abduction and adduction moments at the knee (Hewett et al., 1999). Moments which load the ACL increase the likelihood of tissue damage and rupture during jump landings common in capoeira and a number of other sports. Hewett and colleagues (2006) implemented a six week strength and plyometric training intervention in pre-season female adolescent athletes. These authors noted reduced landing forces, abduction and adduction moments, and in the following year of competition, a significantly lower incidence of ACL injury than in those that did not receive the intervention. In addition to a link between landing forces and risk of traumatic injury, it has been suggested that repetitive landing with high vGRF can lead to stress injuries because of the high impact load (Seegmiller and McCaw, 2003). As capoeira has aspects in common with gymnastics, capoeira athletes may be at a higher risk of lower limb injury due to chronic repetitive high loading.

The present investigation was a pilot study, with three primary limitations. Firstly, the lack of a control group means that the magnitude of change needs to be considered in the context of the potential for some alterations in landing mechanics related to familiarisation with the test rather than CST induced changes alone. Secondly, our study lacks the assessment of kinematics and joint moments. These measurements should be included in future work since peak vGRF in the first landing of a drop jump has been shown to be related to injury prospectively (Hewett et al., 2005), kinematic alterations are also important components of injury risk reduction (Hewett et al., 1999; Hewett et al., 2006). Kinematic data may help to elucidate a mechanism for the kinetic alterations observed. Lastly, the intervention was principally trunk dominant core stability exercises, and the mixture of static and dynamic exercises meant we could not conclude which of these contraction types was responsible for the outcomes observed. Given Imai and colleagues’ (2014) recent findings, showing a distinct reactive jump performance outcome following purely static versus purely dynamic trunk exercise interventions, future work should compare these distinct interventions on vGRF changes.

Our results indicate that CST which includes isometric trunk exercises may be an important component of lower extremity injury prevention programmes and may have contributed to the preferential landing kinetics seen in previous multi-component programs. Given previous evidence indicating that landing kinetics are associated with ACL loading (Hewett et al., 1999; Hewett et al., 2005) these changes may have implications in the design of preventive conditioning programs for non-contact ACL injuries in trained females.

## Electronic Supplementary Material 1

Training intervention demonstrations

## Figures and Tables

**Table 1:** Structure of the six-week training program. Three training sessions were performed per week

**Exercise**	**Weeks 1 and 2**	**Weeks 3 and 4**	**Exercise**	**Weeks 5 and 6**
Plank	*3 × 30 s hold*	*3 × 45 s hold*	One arm plank	*3 × 45 s hold*
Side plank	*3 × 30 s hold*	*3 × 45 s hold*	One arm side plank	*3 × 45 s hold*
Supine bridge	*3 × 30 s hold*	*3 × 45 s hold*	Single leg supine bridge	*3 × 45 s hold*
Abdominal crunch	*3 × 20 repetitions*	*3 × 30 repetitions*	Abdominal crunch	*3 × 45 repetitions*
Russian twist	*3 × 20 repetitions*	*3 × 30 repetitions*	Russian twist	*3 × 45 repetitions*
Split leg scissors	*3 × 20 repetitions*	*3 × 30 repetitions*	Split leg scissors	*3 × 45 repetitions*

**Table 2:** Landing kinetics pre- and post- six week core stability training

	Week 0	Week 6
Weight (N)	591.14 ± 70.81	587.44 ± 56.73
Peak vGRF (first landing phase [N·N_BW_^−1^])	3.40 ± 0.78	2.85 ± 0.52^**^
Peak vGRF (second landing phase [N·N_BW_^−1^])	5.09 ± 1.17	3.02 ± 0.41^**^
Jump height (m)	0.21 ± 0.06	0.21 ± 0.04
Peak loading rate (first landing phase [N·N_BW_·s^−1^])	220.26 ± 111.51	120.27 ± 64.57^*^
Peak loading rate (second landing phase [N·N_BW_·s^−1^])	99.52 ± 54.98	44.71 ± 30.34^*^
Average loading rate (first landing phase [N·N_BW_·s^−1^])	39.85 ± 43.94	23.76 ± 27.89
Average loading rate (second landing phase [N·N_BW_·s^−1^])	30.96 ± 18.84	12.06 ± 9.83^**^

Values are mean ± SD.

* denotes significant difference from week 0 (p<0.01).

** denotes significant difference from week 0 (p<0.001).
