# Supplementary figures and images for: Six Weeks of Core Stability Training Improves Landing Kinetics Among Female Capoeira Athletes: A Pilot Study
Source: J Hum Kinet. 2015 Apr 7;45:27–37. doi: 10.1515/hukin-2015-0004 (PMC4415841; doi:10.1515/hukin-2015-0004)

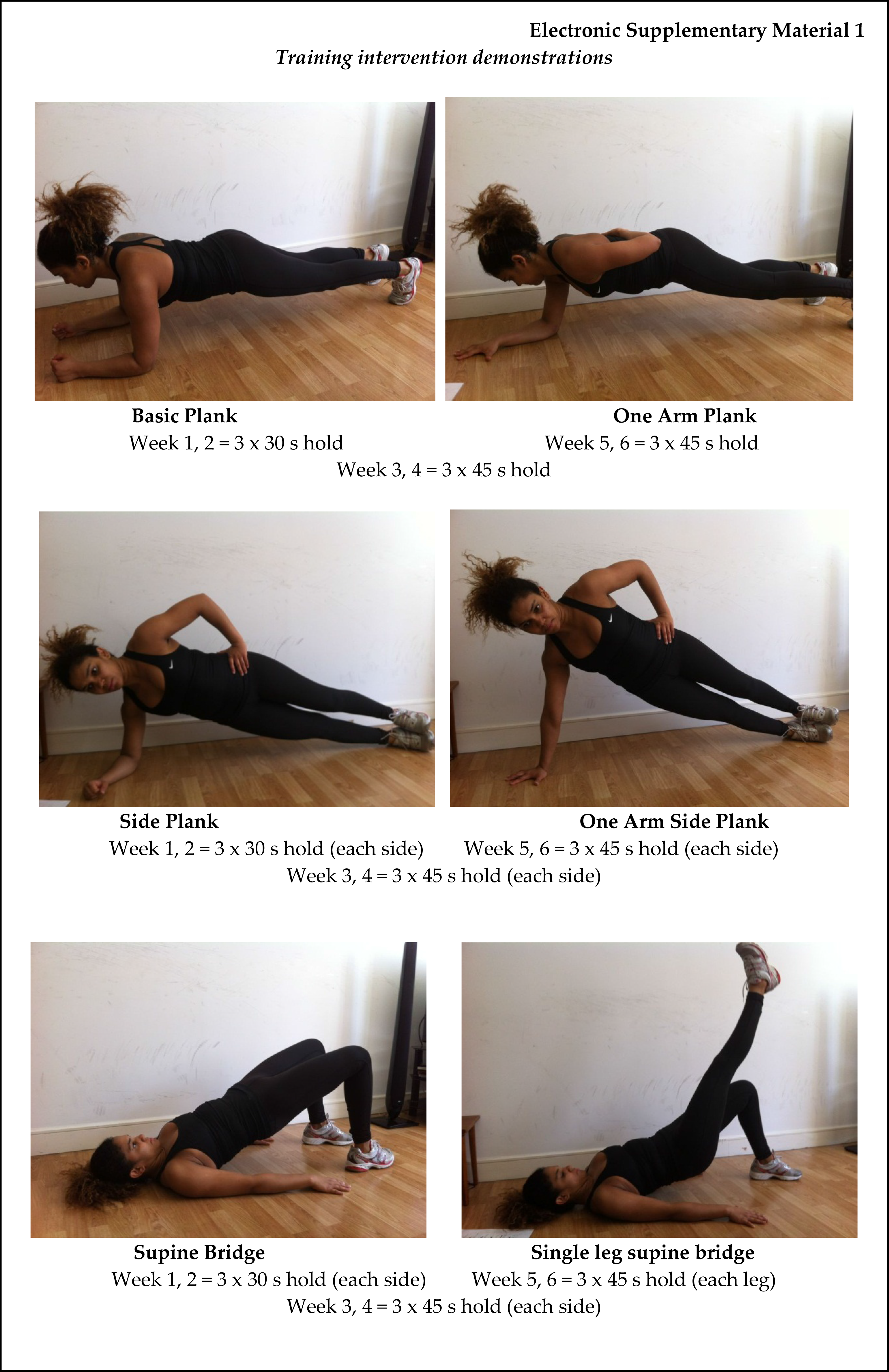

Supplement: Supplementary file 1 [file jhk-45-27f1.tif]

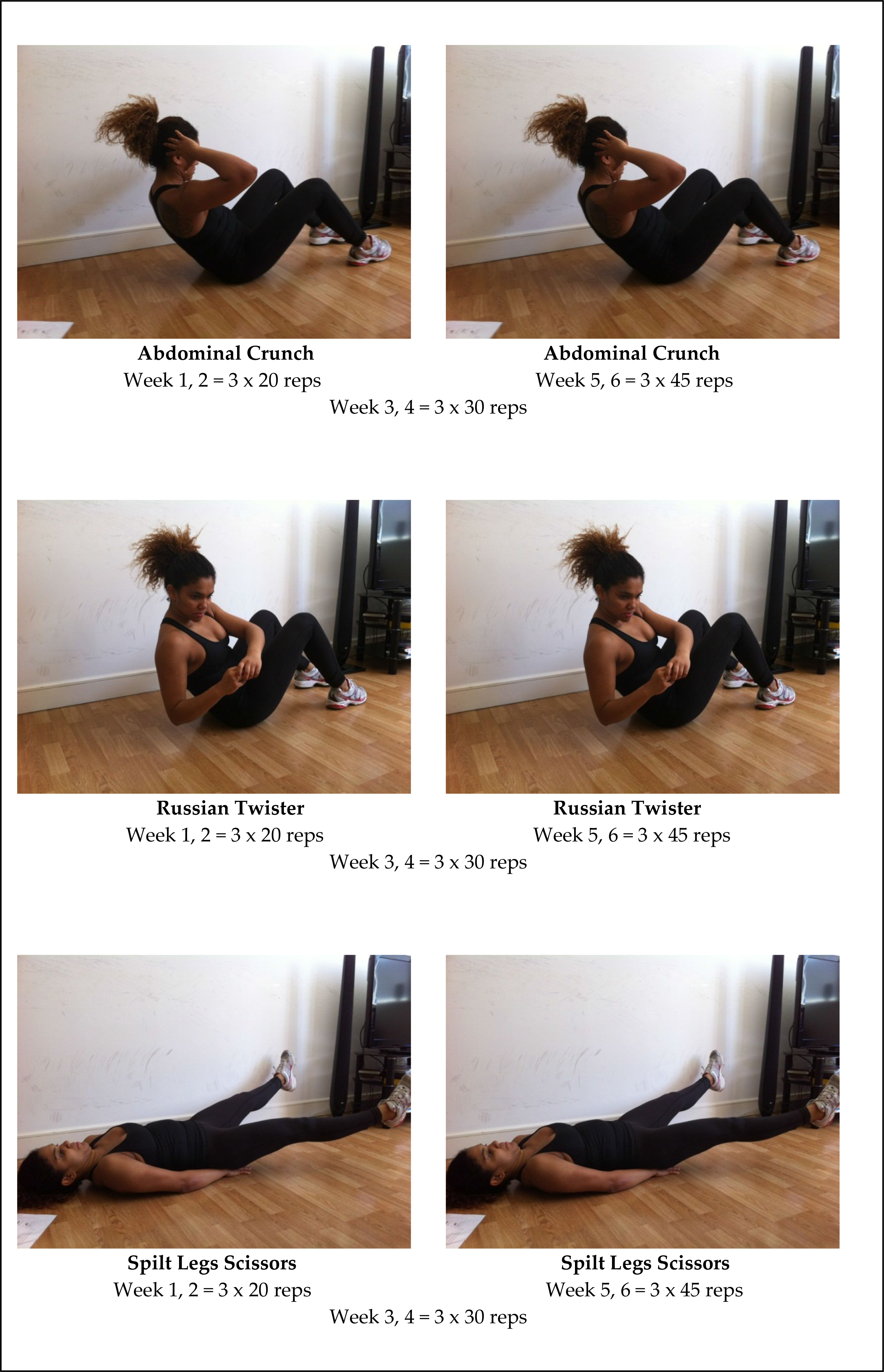

Supplement: Supplementary file 2 [file jhk-45-27f2.tif]
